# MicroRNA expression profiling and Notch1 and Notch2 expression in minimal deviation adenocarcinoma of uterine cervix

**DOI:** 10.1186/1477-7819-12-334

**Published:** 2014-11-08

**Authors:** Heejeong Lee, Kyu Rae Kim, Nam Hoon Cho, Sung Ran Hong, Hoiseon Jeong, Sun Young Kwon, Kwang Hwa Park, Hee Jung An, Tae Heon Kim, Insun Kim, Hye Kyoung Yoon, Kwang Sun Suh, Ki Ouk Min, Hyun Joo Choi, Ji Young Park, Chong Woo Yoo, Youn Soo Lee, Hee Jin Lee, Weon Sun Lee, Chul Soo Park, Yonghee Lee, The Gynecological Pathology Study Group of the Korean Society of Pathologists

**Affiliations:** Department of Hospital Pathology, College of Medicine, The Catholic University of Korea, 222 Banpo-daero, Seocho-gu, Seoul 137-701 Korea; Department of Pathology, Asan Medical Center, University of Ulsan College of Medicine, 388-1, Pungnap 2-dong, Songpa-gu, Seoul 138-736 Korea; Department of Pathology, Yonsei University College of Medicine, 50-1, Yonse-ro, Seodaemun-gu, Seoul 120-752 Korea; Department of Pathology, Cheil General Hospital and Women’s Healthcare Center, Kwandong University College of Medicine, 1-19, Mukjeong-dong, Jung-gu, Seoul 100-380 Korea; Department of Pathology, Keimyung University College of Medicine, 56, Dalseong-ro, Jung-gu, Daegu 700-712 Korea; Department of Pathology, Yonsei University College of Medicine, 20, Ilsan-ro, Wonju, Kangwon-do 220-701 Korea; Department of Pathology, CHA University College of Medicine, 59, Yatap-ro, Bundang-gu, Seongnam, Gyeonggi-do 463-712 Korea; Department of Pathology, Korea University College of Medicine, 73, Inchon-ro, Seongbuk-gu, Seoul 136-705 Korea; Department of Pathology, Inje University College of Medicine, 75, Bokji-ro, Jin-gu, Busan 614-735 Korea; Department of Pathology, Chungnam University College of Medicine, 282, Munhwa-ro, Jung-gu, Daejeon 301-721 Korea; Department of Pathology, Kyungpook National University School of Medicine, 130, Dongdeok-ro, Jung-gu, Daegu 700-721 Korea; Department of Pathology, Center for Uterine Cancer, Research Institute and Hospital, National Cancer Center, 323, Ilsan-ro, Ilsandong-gu, Goyang, Gyeonggi-do 410-769 Korea; Department of Clinical Medicine Research Institute, The Catholic University of Korea, Bucheon St. Mary’s Hospital, 327, Sosa-ro, Wonmi-gu, Bucheon, Gyeonggi-do 420-717 Korea; Department of Internal Medicine, College of Medicine, The Catholic University of Korea, 222, Banpo-daero, Seocho-gu, Seoul 137-701 Korea; Department of Pathology, Ajou University College of Medicine, 164, World Cup-ro, Yeongtong-gu, Suwon, Gyeonggi-do 443-380 Korea

**Keywords:** microRNA, Minimal deviation adenocarcinoma, Notch, Uterine cervix

## Abstract

**Background:**

MicroRNA (miRNA) expression is known to be deregulated in cervical carcinomas. However, no data is available about the miRNA expression pattern for the minimal deviation adenocarcinoma (MDA) of uterine cervix. We sought to detect deregulated miRNAs in MDA in an attempt to find the most dependable miRNA or their combinations to understand their tumorigenesis pathway and to identify diagnostic or prognostic biomarkers. We also investigated the association between those miRNAs and their target genes, especially Notch1 and Notch2.

**Methods:**

We evaluated miRNA expression profiles via miRNA microarray and validated them using.real-time PCR assays with 24 formalin-fixed, paraffin-embedded tissue blocks of MDA and 11 normal proliferative endocervical tissues as control. Expression for Notch1 and 2 was assessed by immunohistochemistry.

**Results:**

MiRNA-135a-3p, 192-5p, 194-5p, and 494 were up-regulated, whereas miR-34b-5p, 204-5p, 299-5p, 424-5p, and 136-3p were down-regulated in MDA compared with normal proliferative endocervical tissues (all *P* <0.05). Considering the second-order Akaike Information Criterion consisting of likelihood ratio and number of parameters, miR-34b-5p showed the best discrimination power among the nine candidate miRNAs. A combined panel of miR-34b-5p and 194-5p was the best fit model to discriminate between MDA and control, revealing 100% sensitivity and specificity. Notch1 and Notch2, respective target genes of miR-34b-5p and miR-204-5p, were more frequently expressed in MDA than in control (63% vs. 18%; 52% vs. 18%, respectively, *P* <0.05). MiR-34b-5p expression level was higher in Notch1-negative samples compared with Notch1-positive ones (*P* <0.05). Down-regulated miR-494 was associated with poor patient survival (*P =*0.036).

**Conclusions:**

MDA showed distinctive expression profiles of miRNAs, Notch1, and Notch2 from normal proliferative endocervical tissues. In particular, miR-34b-5p and 194-5p might be used as diagnostic biomarkers and miR-494 as a prognostic predictor for MDA. The miR-34b-5p/Notch1 pathway as well as Notch2 might be important oncogenic contributors to MDA.

**Electronic supplementary material:**

The online version of this article (doi:10.1186/1477-7819-12-334) contains supplementary material, which is available to authorized users.

## Background

Minimal deviation adenocarcinoma (MDA), also known as adenoma malignum, is an extremely well-differentiated variant of cervical adenocarcinoma in which most of the cells lack the cytological features of malignancy [1]. Although MDA is an uncommon tumor and accounts for only 1 to 3% of all cervical adenocarcinomas [1], it deserves scrutiny because many non-neoplastic endocervical glandular lesions encountered in daily practice should be differentiated from it. Because it is very difficult to diagnose preoperatively if the proliferative endocervical glandular lesion is definitely benign or malignant, especially in punch biopsy specimens [2], it would be crucial to combine ancillary molecular or immunohistochemical biomarkers with morphologic characteristics in order to improve diagnostic accuracy. In 1998, a Japanese group initially demonstrated that MDA showed immunoreactivity with HIK1083 [3], and ever since this antibody has been considered a promising tool for establishing the diagnosis of MDA. However, its usefulness has been in dispute because its immunoreactivity has also been demonstrated in several proliferative endocervical glandular lesions exhibiting gastric differentiation, including lobular endocervical glandular hyperplasia (LEGH) or gastric-type adenocarcinoma in situ (AIS), as well as in MDA [4, 5].

Whereas most cases of cervical carcinomas are known to be associated with high-risk human papillomavirus infection, MDA is usually found to be negative for human papillomavirus [1, 6, 7]. Although some authors suggested that MDA could be derived from LEGH and morphologically usual-type AIS showing gastric immunophenotype [1, 4], further studies would be needed to draw this conclusion. Previous reports for prognosis of MDA have not provided uniform data. Several investigators reported poor prognosis [8–10], whereas some found a relatively favorable prognosis similar to that for other well-differentiated cervical adenocarcinomas [11–13]. Therefore, difficulty in morphologic diagnosis, uncertain pathogenesis, and variable clinical outcome data prevent clinicians from guiding patient clinical management and treatment effectively.

MicroRNAs (miRNAs) are small non-coding RNAs that have been implicated in tumor development [14, 15]. They regulate target gene expression either by mRNA degradation or by translation repression [14–18]. In general, each miRNA can regulate up to hundreds of target genes [14–18]. During tumor development, aberrant expression of miRNAs can either inactivate tumor suppressor genes or activate oncogenes, thereby promoting tumor formation [14–16, 18, 19]. Because expression of miRNAs is tissue-specific [14, 15], detectable in blood [20], and correlates with clinical cancer behaviors [21], miRNAs are potentially valuable biomarkers.

Until recently, several studies have identified either up-regulated or down-regulated miRNAs in cervical cancers using the global profiling method [22–29] or the candidate miRNA approach [30]. However, most studies determined miRNA expression profiles in cervical squamous cell carcinomas [22–30], although a few adenocarcinoma cases were included in the cervical cancer category. There has been no report about the miRNA expression profiles on an independent set of cervical adenocarcinomas, in particular, MDA.

Characterization of the complex relationship between deregulated miRNAs and their target genes in MDA may not only help to define some of the molecular pathways that drive carcinogenesis, but those biomarkers would also help to improve discrimination between MDA and other glandular lesions and be of considerable importance for the prediction of prognosis.

## Methods

### Tissue samples and patients

A total of 24 archived formalin-fixed paraffin-embedded (FFPE) tissue blocks of MDA, excluding cases associated with Peutz-Jeghers syndrome, were obtained from the pathology department of 13 hospitals in Korea. A central review with hematoxylin-eosin-stained slides was undertaken by at least 10 pathologists from the Gynecological Pathology Study Group of the Korean Society of Pathologists. Of 24 carcinomas, 11 cases were in stage Ib, 8 in stage II, 1 in stage III, and 2 in stage IV, according to the International Federation of Gynecology and Obstetrics standards. A total of 11 cases of normal proliferative endocervical tissue (NE) were obtained from the patients who had a hysterectomy for benign uterine pathologies, such as adenomyosis or leiomyoma. For minimal deviation adenocarcinoma, we selected only the mucinous variant of MDA since they are the main targets and discrimination from various non-neoplastic proliferative endocervical lesions as well as normal endocervix must be made. Only tissue blocks with more than 70% carcinoma content were used for this study. The clinicopathological characteristics of the patients are summarized in Table 1. This study protocol was approved by the Institutional Review Board of Bucheon St. Mary’s Hospital from the Catholic University of Korea.

**Table 1:** **Clinicopathological characteristics of patients with minimal deviation adenocarcinoma (n =24)**

Characteristics	No. (%)
Age (median, range; yrs)	46 (30–59)
Tumor size	
≤4 cm	15 (62.5)
>4 cm	9 (37.5)
Stage	
Low (I/II)	21 (87.5)
High (III/IV)	3 (12.5)
Lymph node involvement	
Absent	18 (75.0)
Present	6 (24.0)
Distant metastasis	
Absent	20 (83.3)
Present	4 (16.7)
Overall survival (range; months)	4–135

### RNA extraction

Total RNA was extracted from FFPE tissues using an miRNeasy FFPE kit (Qiagen, Hilden, Germany) following the manufacturer’s instructions. RNA concentration and purity were assessed using a UV spectrophotometer.

### MicroRNA expression profiling assay

For the selection of the candidate miRNAs, we evaluated miRNA expression profiles of 8 MDA and 8 NE samples which were randomly selected. The miRNA Microarray System with miRNA Complete Labeling and Hybridization kit (SurePrint G3 Human miRNA Microarray, Release 18.0, 8×60K, Agilent Technologies, Santa Clara, CA, USA) containing 1,887 human miRNA oligonucleotide probes was used according to the manufacturer’s recommended protocol. The Agilent microRNA Spike-In kit was used for in-process control to measure labeling and hybridization efficiency. Arrays were scanned on an Agilent Technologies G4900DA SureScan scanner using 3-μm resolution. RNA hybridization and scanning were performed by Macrogen Inc. (Seoul, Korea).

### Reverse transcription and quantitative real-time PCR

Reverse transcription and quantitative real-time PCR (qRT-PCR) were performed for the validation of the selected miRNAs using the MicroRNA TaqMan® Reverse Transcription Kit and the TaqMan MicroRNA Assays in triplicate (Applied Biosystems, Foster City, CA, USA). U6 small nuclear 2 (RNU6b) was used to normalize input total small RNA. Absolute quantification for each miRNA as well as RNU6b was performed using a standard curve generated by serial dilution of reverse-transcribed total RNA extracted from VK2 cells, and expression of each miRNA was presented as the ratio between miRNA and RNU6b (RQ).

### Immunohistochemical analysis

Immunohistochemistry for Notch1 (Cell Signaling Technology, #3608, Danvers, MA, USA; dilution 1:100) and Notch2 (sc-5545, Santa Cruz Biotechnology, Santa Cruz, CA, USA; dilution 1:100) were performed using standard staining procedures as described previously [31]. All cases were reviewed and interpreted without knowledge of other laboratory or clinical results. Immunohistochemical reactions were categorized simply as positive or negative because of the small number of tissue samples.

### Raw data preparation and statistical analysis

Raw data from the microarray analysis were extracted using the Agilent Feature Extraction Software (v11.0.1.1). The array data were filtered by “gIsGeneDetected” =1 in all samples (1: detected). The selected “miRNAgtotalGeneSignal” value was logarithmically transformed and normalized by the quantile method. A comparative analysis between the test and control samples was carried out using fold-change and an independent *t*-test. The false discovery rate was controlled by adjusting *P* value using the Benjamini-Hochberg algorithm. Hierarchical cluster analysis was performed using complete linkage and Euclidean distance as a measure of similarity.

The RQ values in qRT-PCR data were logarithmically transformed due to highly skewed distribution of RQ levels. The Mann–Whitney test and χ^2^ test were used to compare the miRNA, Notch1, and Notch2 expression between MDA and NE specimens. To determine the correlation between miRNAs and pathological diagnoses, we conducted a Firth’s bias reduced logistic regression analysis [32] to reduce the bias due to “separation” and significant multicolinearties between miRNAs. The best-fit model was determined by the second order Akaike Information Criterion (AICc). The receiver-operating characteristic (ROC) curves were constructed; the sensitivity and specificity at each cut-off value and area under the ROC curve (AUC) were estimated. The correlation between two most down-regulated miRNAs and their target genes, Notch1 and Notch2, and the correlation between miRNA or Notch expressions and clinicopathologic parameters were evaluated with the Mann–Whitney test. Survival curves were produced via the Kaplan-Meier method and the resulting curves were compared using the log-rank test. *P* values <0.05 were considered statistically significant. Due to a small number of cases, we could not derive strong correlation between miRNAs, Notch 1, Notch 2, and clinicopathologic parameters. Statistical analysis was performed using R statistical language v. 2.15.0 and SPSS 17.0.

## Results

### Results of microRNA microarray analysis

**Figure 1:**
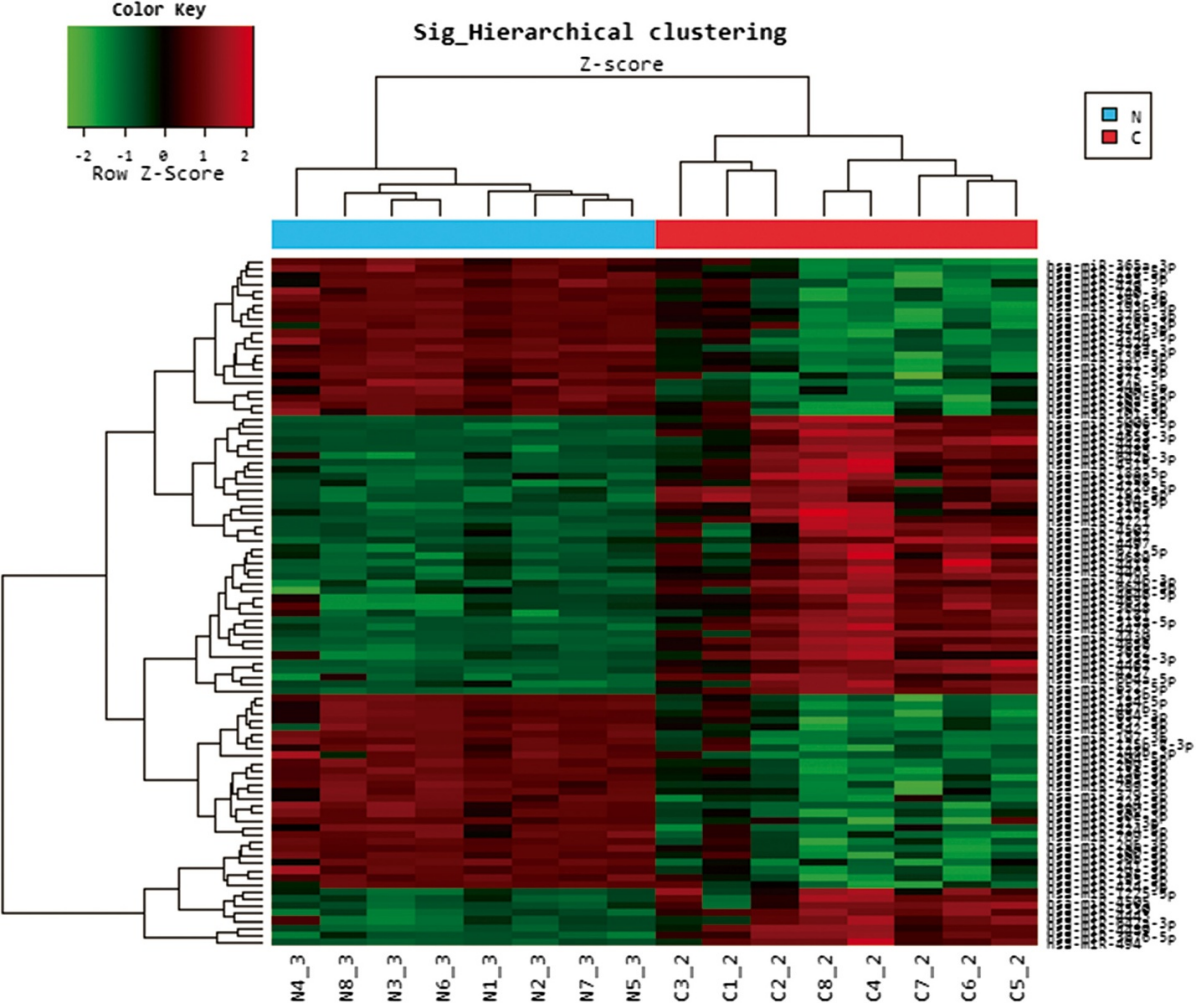
**Unsupervised hierarchical clustering analysis based on miRNA array data.** Array data from 8 minimal deviation adenocarcinomas (MDA) (C1–C8) and 8 normal proliferative endocervical tissues (NE) (N1–N8) shows 47 overexpressed miRNAs (red) and 49 underexpressed miRNAs (green) in MDA compared to NE.

We examined the expression levels of miRNAs in MDA (n *=*8) and NE (n *=*8), for a total of 16 independent samples. Using unsupervised hierarchical clustering analysis without any information on the identity of the samples, a tree was generated that represented a clear separation of MDA from NE (Figure 1). Among 1,887 miRNAs analyzed, there was a significant difference in the expression level of 96 miRNAs (47 up-regulated and 49 down-regulated) with more than a three-fold change between MDA and NE. Among these, the most significantly overexpressed miRNAs in MDA were miR-494, 135a-3p, 513a-5p, 194-5p, 192-5p, and 188-5p, whereas the most significantly down-regulated miRNAs were miR-34b-5p, 204-5p, 299-5p, 424-5p, and 136-3p.

### Results of validation study on reverse transcription and quantitative real-time PCR

**Figure 2:**
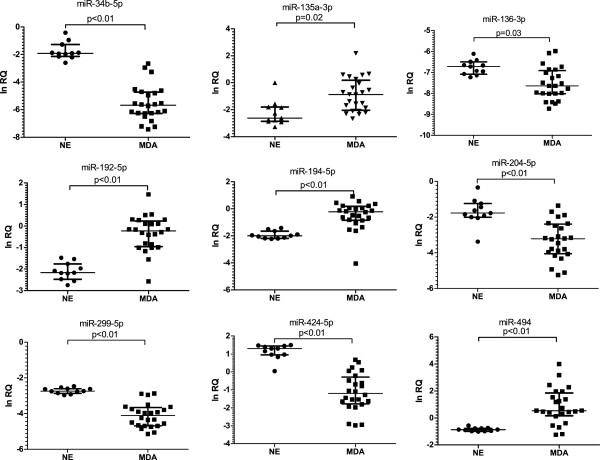
**Validation of the differentially-expressed miRNAs from the microarray data with real-time quantitative PCR.** Expression of each miRNA in a validation set composed of normal proliferative endocervical tissues (NE) and minimal deviation adenocarcinoma (MDA) of uterine cervix is shown by an individual scatter plot.

To validate the data from the miRNA microarray, we used qRT-PCR to analyze the expression levels of the six up-regulated and five down-regulated candidate miRNAs, using a set of MDA (n *=*24) and NE (n *=*11), including the samples used for microarray analysis. Expression levels of four miRNAs (miR-494, 135a-3p, 194-5p, and 192-5p) were significantly higher and those of five miRNAs (miR-34b-5p, 204-5p, 299-5p, 424-5p, and 136-3p) were significantly lower in MDA when compared with NE (*P* <0.05 for both; Figure 2). Through a validation study, we found that miR-513a-5p and miR-188-5p were not significantly up-regulated in MDA compared to control, unlike the microarray data. Thus, we discarded those two miRNAs and chose only the nine miRNAs which were confirmed by real-time PCR.

### MicroRNAs as biomarkers for detecting minimal deviation adenocarcinoma

The individual miRNAs exhibited a significant correlation with MDA in univariate logistic regression analysis with AICc values ranging from 10.069 to 38.194 (all *P* <0.05). The best single miRNA to discriminate MDA from NE was miR-34b-5p with an AICc value of 10.069 (Table 2). To discriminate MDA from NE samples, the composite panel of two miRNAs (miR-34b-5p and 194-5p) was determined to be the best fitting model, using Firth’s bias reduced multivariate logistic regression analysis. The following regression equation was built: Logit (P) = -4.068 – 1.900* (ln miR-34b-5p) +1.396* (ln miR-194-5p). The odds ratios of ln miR-34b-5p and 194-5p were 0.149 and 4.035, respectively, and this model exhibited an AICc value of 8.190, which is lower than that of any single miRNA (Table 2).

**Table 2:** **Univariate and multivariate logistic regression analysis result and AICc values for differentiating MDA from NE**

ln miRNAs	OR	95 % CI	***P***value	AICc value
miR-34b-5p	0.012	0.002–0.358	<0.001	10.069
miR-135a-3p	3.938	1.348–11.510	0.012	36.902
miR-136-3p	0.144	0.032–0.046	0.011	38.194
miR-192-5p	31.808	2.912–347.453	0.005	20.098
miR-194-5p	7.921	1.933–32.459	0.004	31.019
miR-204-5p	0.133	0.031–0.561	0.006	30.576
miR-299-5p	0.006	0.000 – 0.116	0.002	16.953
miR-424-5p	0.011	0.000–0.411	0.015	15.014
miR-494	26.278	1.861–370. 966	0.016	25.869
**Best fit**				
ln miR-34b-5p	0.149	0.008–0.453	<0.001	
ln miR-194-5p	4.035	1.150–50.958	0.032	
constant	0.017	0.000–0.662	0.027	8.190

MDA, Minimal deviation adenocarcinoma; NE, Normal proliferative endocervical tissue; OR, Odds ratio; CI, Confidence interval; AICc, Second-order Akaike Information Criterion.

The individual miRNAs exhibited AUC values of 0.814 to 1.000 in distinguishing MDA from NE, revealing 70.8 to 100% sensitivity and 81.8 to 100% specificity (all *P* <0.05, Table 3). The best fitting model consisting of miR-34b-5p and 194-5p produced an AUC value of 1.000, 100% sensitivity, and 100% specificity (*P* <0.01) (Table 3).

**Table 3:** **Capabilities of the ln miRNAs to discriminate MDA from NE**

ln miRNAs	AUC (SE)	Sensitivity %	Specificity %	***P***value
miR-34b-5p	1.000 (0.000)	100.0	100.0	0.000
miR-135a-3p	0.833 (0.078)	70.8	81.8	0.002
miR-136-3p	0.814 (0.072)	70.8	90.9	0.003
miR-192-5p	0.955 (0.039)	91.7	100.0	0.000
miR-194-5p	0.943 (0.043)	95.8	81.8	0.000
miR-204-5p	0.886 (0.060)	80.3	90.9	0.000
miR-299-5p	0.981 (0.013)	91.6	90.9	0.000
miR-424-5p	0.981 (0.021)	100.0	90.9	0.000
miR-494	0.909 (0.057)	91.7	90.9	0.000
2 miRNAs*	1.000 (0.000)	100.0	100.0	0.000

MDA, Minimal deviation adenocarcinoma; NE, Normal proliferative endocervical tissue; AUC, Area under receiver-operating characteristics curve; SE, Standard error.

*Model is constructed using Firth’s bias reduced logistic regression analysis.

All *P* <0.05.

### Selected microRNAs and computational analysis for their predicted target genes

We identified the predicted target genes via web-based computational approaches (miRDB; http://mirdb.org, ver 4.0, miRBase rel.18.0). We discovered that Notch1 and Notch2 were the target genes of the most down-regulated miRNAs, miR-34b-5p and miR-204-5p, respectively.

### Notch1 and Notch2 status and association with microRNA expression

**Figure 3:**
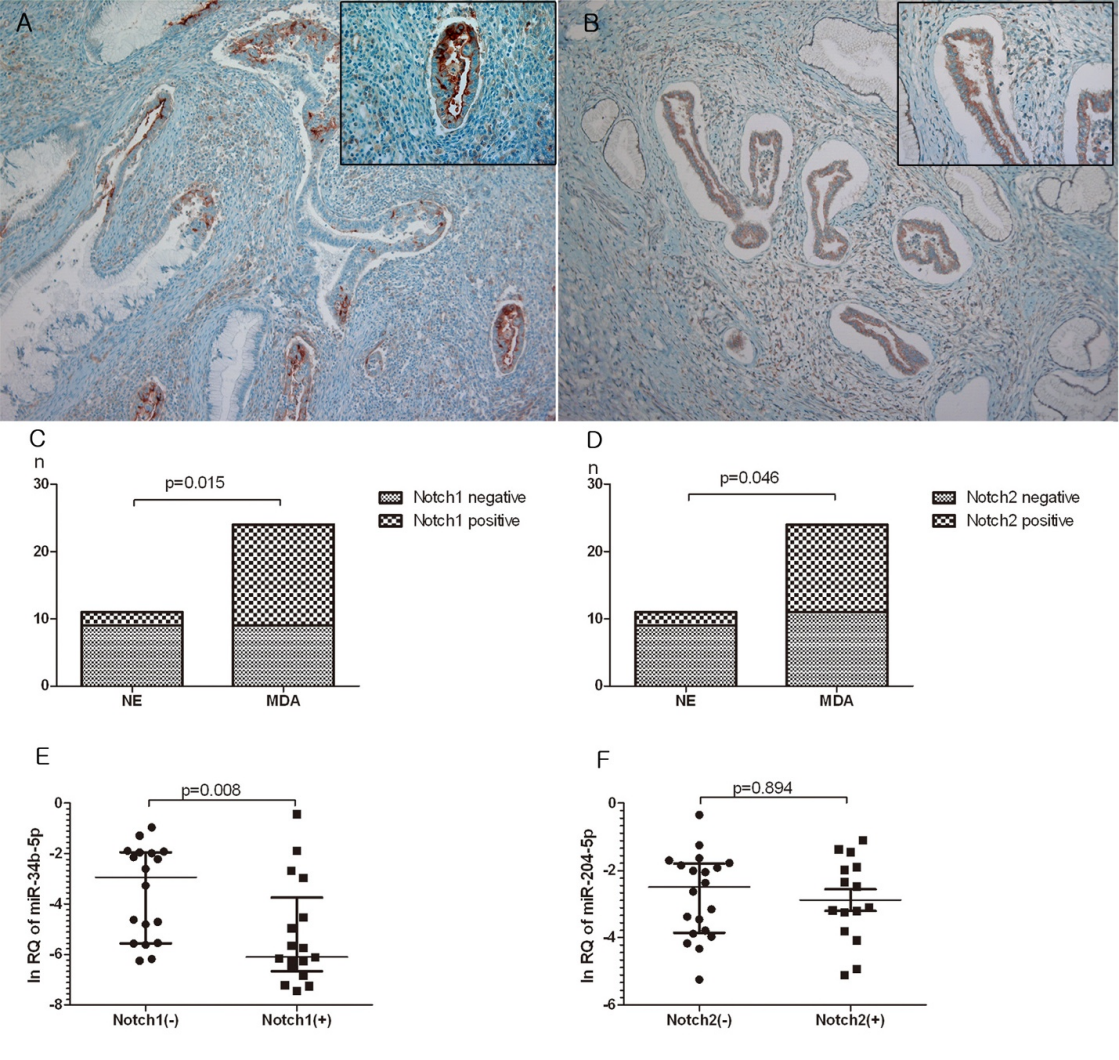
**Immunohistochemical staining of Notch1 and Notch2 in minimal deviation adenocarcinoma (MDA) of uterine cervix and their correlation with miRNAs.** **(A)** Notch1 is predominantly localized at the apical membranes and **(B)** Notch2 is expressed in the cytoplasm of the tumor cells. High magnification view (inset, ×400). Differential expression of **(C)** Notch1 and **(D)** Notch2 in MDA compared to normal proliferative endocervical tissue (NE). Expression levels of **(E)** miR-34b-5p and **(F)** miR-204-5p according to Notch1 and Notch2 status, respectively.

Notch1 and Notch2 expressions were determined by immunohistochemical analysis (Figure 3A, B) in the same set of MDA (n *=*24) and NE (n *=*11) used for qRT-PCR. Notch1 was detected in 11 of 24 MDA (63%) and 2 of 11 NE (18%) samples, whereas Notch2 was detected in 13 of 24 MDA (52%) and 2 of 11 NE (18%) samples. The percentages of positive Notch1 and Notch2 samples were significantly higher in MDA compared with NE samples (63% vs. 18%, *P* =0.015 and 52% vs. 18%, *P* =0.046, respectively; Figure 3C, D). Expression of miR-34b-5p was significantly up-regulated in Notch1-negative samples compared with Notch1-positive samples (*P* =0.008; Figure 3E). However, miR-204-5p did not show a significant correlation with the Notch2 expression pattern (*P* =0.894; Figure 3F).

### Association with clinicopathological parameters and survival

**Figure 4:**
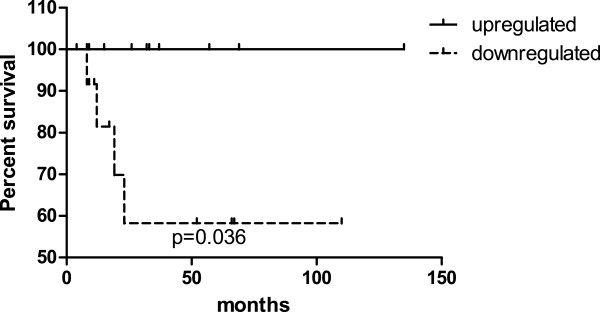
**Kaplan-Meier survival curve and log-rank test for miR-494 level.** The survival rate of patients with down-regulated miR-494 levels was significantly lower than that of patients with up-regulated miR-494 levels (log-rank test *P* =0.036).

Down-regulation of miR-494 was associated with poor patient survival (*P* =0.036; Figure 4). We found no significant association between miRNA expression and tumor size, clinical stage, lymph node, or distant metastasis, nor between Notch expression and various clinicopathological parameters (see Additional file 1).

## Discussion

Preoperative differential diagnosis of MDA from other proliferative glandular lesions, such as endocervical tunnel clusters [1], deeply situated nabothian cysts [1], endocervicosis of the cervical wall [1], mesonephric hyperplasia [1], LEGH [2], and AIS [2], is critical for appropriate therapeutic management. Nonetheless, according to a previous report [2], the interobserver agreement rate for MDA showed just a slight level of consistency (k-value =0.115).

Recently, several studies have noted associations between microRNA expression and cervical carcinomas [22–30]. MiRNAs reported in the previous studies [22–30] to be associated with cervical carcinomas included the following: miR-21, 143, 21, 145, 218, 29a, 155, 16, 146a, 20a, 126, 127, 424, 17-5p, 203, 20, 15b, 106a, 148a, 224, 10b, 450, 199a, 20b, 125b, 15a, 93, 182, 185, and 34a. However, those studies revealed quite different miRNA expression profiling patterns from ours because most studies have focused on cervical squamous cell carcinomas. These findings are explained by the previous reports that have suggested tissue-specific miRNA expression patterns with different sets of miRNAs up- or down-regulated in tumors of different cellular origin [33–35].

In the present study, using miRNA microarray and qRT-PCR on 24 MDA samples, we detected nine miRNAs which were differently expressed between MDA and NE. They exhibited relatively good discrimination ability and, in particular, single miR-34b-5p testing could discriminate MDA from NE with 100% sensitivity and specificity. We also constructed the best fitting model consisting of miR-34b-5p and 194-5p based on AICc values, and it also showed 100% sensitivity and specificity. Considering the high sensitivity and specificity above 99%, they might be used as supplementary diagnostic biomarkers in pathologically complicated cases with ambiguous morphological features or with only small amounts of superficial glandular lesions upon limited sampling.

Four Notch genes have been described in mammals, Notch1, Notch2, Notch3, and Notch4 [31, 36, 37]. They encode type I transmembrane proteins with extracellular domains containing epidermal growth factor-like repeats that regulate cell proliferation and differentiation in various tissues [31, 36, 37]. In cervical cancer, upregulation of Notch1 [37–39] and Notch2 [39] was associated with in-situ or invasive squamous cell carcinoma and adenocarcinoma, and it is thought that abnormal Notch signaling can promote the development of cervical cancer [40]. In the present study, Notch1 and Notch2 seemed to play an oncogenic role because both of them were significantly up-regulated in MDA compared to normal control. Interestingly, although we observed that Notch1 expression was dependent on the miR-34b-5p expression level, Notch2 expression did not seem to be dependent on the miR-204-5p expression level, thereby suggesting another mechanism or miRNAs might be involved in the Notch2 expression process in the MDA carcinogenesis pathway. In particular, considering that miR-34b-5p and Notch1 showed the most distinctive expression pattern between MDA and NE, we can speculate that down-regulation of miR-34b-5p and the resulting overexpression of the Notch1 gene (miR-34b-5p/Notch1 pathway) might comprise one of the important oncogenic pathways of MDA. However, Notch2 is positive in 52% of MDAs compared to 18% of normal control samples, and this finding alone may be sufficient to suspect that Notch2 could also play a role in carcinogenesis of MDA, although maybe not triggered by miR-204-5p.

To date, several miRNAs, including miR-375 [41], 127 [24], 9 [42], 200a [42, 43], 93 [43], 497 [44], and 224 [45], have been reported to be associated with metastasis, progression, or survival of cervical cancer, although most of the cervical tumors in those studies were also squamous cell type. In this study, down-regulation of miR-494 was associated with poor patient survival, suggesting its possible role as a prognostic marker.

Limitations to our study need to be mentioned. First, even though we performed a multicenter study, there was still only a small number of cases due to the low incidence of MDA. Thus, we could not afford another validation study for our diagnostic or prognostic biomarkers with a test set, and future studies with larger sample size, notably including controversial cases, will have to be performed. Second, a possible precursor lesion, like LEGH or gastric type-AIS, which could strengthen our hypothesis was not included in this study. Third, more experiments, such as transfection of miR-34b-5p and cell viability tests, are still warranted to reveal the exact role of the miR-34b-5p/Notch1 pathway during MDA carcinogenesis.

## Conclusions

MDA showed distinctive expression profiles of miRNAs, Notch1, and Notch2 from NE. In particular, miR-34b-5p and 194-5p might be used as diagnostic biomarkers and miR-494 as a prognostic predictor for MDA. The miR-34b-5p/Notch1 pathway as well as Notch2 might be important oncogenic contributors to MDA.

## Electronic supplementary material

Additional file 1: **Correlations between miRNA or Notch expressions and clinicopathological parameters.** (DOCX 24 KB)

## Abbreviations

AICc: Akaike Information Criterion
AIS: Adenocarcinoma in situ
AUC: Area under the ROC curve
FFPE: Formalin-fixed paraffin-embedded
LEGH: Lobular endocervical glandular hyperplasia
MDA: Minimal deviation adenocarcinoma
miRNAs: MicroRNAs
NE: Normal proliferative endocervical tissue
PCR: Polymerase chain reaction
qRT-PCR: Reverse transcription and quantitative real-time PCR
RNU6b: U6 small nuclear 2
RQ: Ratio between miRNA and RNU6b
ROC: Receiver-operating characteristic.
