# Supplementary material for: MicroRNA expression profiling and Notch1 and Notch2 expression in minimal deviation adenocarcinoma of uterine cervix
Source: World J Surg Oncol. 2014 Nov 8;12:334. doi: 10.1186/1477-7819-12-334 (PMC4239392; doi:10.1186/1477-7819-12-334)
Supplement: Supplementary file 1 — Additional file 1: Correlations between miRNA or Notch expressions and clinicopathological parameters. (DOCX 24 KB) [file 12957_2014_1807_MOESM1_ESM.docx]

**Additional file 1** **Correlations between miRNA or Notch expressions and clinicopathological parameters**

| **parameters** | **Tumor size** | | **FIGO stage** | | **Lymph node metastasis** | | **Distant metastasis** | |
| --- | --- | --- | --- | --- | --- | --- | --- | --- |
|  | **Small**  **(n=15)** | **Large**  **(n=9)** | **Low**  **(n=21)** | **High**  **(n=3)** | **Negative**  **(n=18)** | **Positive**  **(n=6)** | **Negative**  **(n=20)** | **Positive**  **(n=4)** |
| ln miR-34-b | -5.619 | -6.185 | -5.619 | -6.276 | -5.674 | -5.898 | -5.682 | -5.906 |
| *P* value |  | 0.421 |  | 0.074 |  | 0.739 |  | 0.877 |
| ln miR-135a-3p | -1.566 | -0.723 | -0.863 | -1.372 | -1.189 | -0.626 | -0.872 | -0.870 |
| *P* value |  | 0.144 |  | 0.896 |  | 0.317 |  | 0.588 |
| ln miR-136-3p | -7.638 | -7.644 | -7.638 | -8.010 | -7.711 | -6.792 | -7.579 | -7.894 |
| *P* value |  | 0.881 |  | 0.631 |  | 0.205 |  | 0.535 |
| ln miR-192-5p | -0.159 | -0.286 | -0.163 | -0.379 | -0.303 | 0.055 | -0.241 | -0.006 |
| *P* value |  | 0.612 |  | 0.827 |  | 0.317 |  | 0.588 |
| ln miR-194-5p | -0.527 | 0.012 | -0.185 | -0.639 | -0.216 | -0.237 | -0.367 | -0.034 |
| *P* value |  | 0.245 |  | 0.631 |  | 0.463 |  | 0.439 |
| ln miR-204-5p | -3.184 | -3.799 | -3.184 | -4.075 | -3.192 | -3.622 | -3.220 | -3.471 |
| *P* value |  | 0.270 |  | 0.127 |  | 0.739 |  | 0.757 |
| ln miR-299-5p | -3.974 | -4.343 | -3.974 | -4.691 | -4.295 | -3.886 | -3.974 | -4.517 |
| *P* value |  | 0.612 |  | 0.256 |  | 0.505 |  | 0.510 |
| ln miR-424-5p | -1.098 | -1.532 | -1.098 | -1.654 | -1.363 | -0.919 | -1.075 | -1.954 |
| *P* value |  | 0.531 |  | 0.315 |  | 0.894 |  | 0.163 |
| ln miR-494 | 0.534 | 0.546 | 0.534 | 1.375 | 0.961 | 0.386 | 0.639 | 0.386 |
| *P* value |  | 0.788 |  | 0.359 |  | 0.286 |  | 0.439 |
| Notch 1 |  |  |  |  |  |  |  |  |
| Negative n(%) | 5(33) | 4(44) | 9(43) | 0(0) | 7(39) | 2(33) | 6(30) | 3(75) |
| Positive n(%) | 10(67) | 5(56) | 12(57) | 3(100) | 11(61) | 4(67) | 14(70) | 1(25) |
| *P* value |  | 0.586 |  | 0.151 |  | 0.808 |  | 0.090 |
| Notch 2 |  |  |  |  |  |  |  |  |
| Negative n(%) | 5(33) | 6(67) | 10(48) | 1(33) | 8(44) | 3(50) | 8(40) | 3(75) |
| Positive n(%) | 10(67) | 3(33) | 11(52) | 2(67) | 10(56) | 3(50) | 12(60) | 1(25) |
| *P* value |  | 0.113 |  | 0.642 |  | 0.813 |  | 0.200 |
